# Knowledge, Awareness and Practice towards Screening for Prostate Cancer: A Systematic Review and Meta-Analysis

**DOI:** 10.4314/ejhs.v33i3.19

**Published:** 2023-05

**Authors:** Adithya G Rao, Winniecia Dkhar, S Sharath, Rajagopal Kadavigere, Abhimanyu Pradhan

**Affiliations:** 1 Department Medical Imaging Technology, Manipal College of Health Professions, Manipal Academy of Higher Education, Manipal – 576104, Karnataka, India. Email:; 2 Department Medical Imaging Technology, Manipal College of Health Professions, Manipal Academy of Higher Education, Manipal – 576104, Karnataka, India; 3 Department Medical Imaging Technology, Manipal College of Health Professions, Manipal Academy of Higher Education, Manipal – 576104, Karnataka, India; 4 Department of Radio diagnosis and Imaging, Kasturba Medical College, Manipal Academy of Higher Education, Manipal – 576104, Karnataka, India; 5 Department Medical Imaging Technology, Manipal College of Health Professions, Manipal Academy of Higher Education, Manipal – 576104, Karnataka, India

**Keywords:** Knowledge, Awareness, Screening, Prostate cancer

## Abstract

**Background:**

Globally prostate cancer is one of the most prevalent cancer among men and a leading cause of morbidity and mortality, especially in a developing country, which is mainly due to lack of knowledge and awareness regarding the screening of prostate cancer. The main objective of this review and meta-analysis is to evaluate the knowledge, awareness and practice of adult men about prostate cancer.

**Method:**

An extensive literature search was performed on studies published between January 2000 to 2021. The systematic review initially yielded 137 studies, out of which 7 studies were covered on this meta-evaluation.

**Result:**

We noted that the pooled estimate of knowledge and awareness were respectively 65% [CI: 29%, 100%], and 74% [CI: 66%, 82%] about prostate cancer. However, there were limited practices noted in screening of prostate cancer.

**Conclusion:**

In order to increase the awareness and screening practice rate for prostate cancer, an improved health education is highly recommended.

## Introduction

Prostate Cancer (prophylaxis-related cancer of the prostate) is one of the most frequent and most prevalent cancer in men worldwide and a leading cause of cancer-related morbidity and mortality. In accordance with the aging process, the probability of developing prostate cancer increases as one ages. Prostate cancer is more likely to develop among those older than 39 years of age, increasing to 2.2% (1 in 45) for those between the ages of 40 and 59 years and 13.7% (1 in 7) for those ages 60 to 79 years. Overall, 16.7% of men will develop prostate cancer in their lifetime (1 in 6). As per GLOBACON census report of 2020, the number of new cases of Prostate cancer are 14,259 and 3, 74,304 deaths have been recorded in the world. It has been reported that prostate cancer is the third most frequently occurring cancer which also stands eight in terms of mortality (1). In India 34,540 new cases of prostate cancer have been reported with 16,783 deaths. The incidence of prostate cancer has been increasing during the last decades especially in industrialised countries like India (2).

The advanced prostate cancer symptoms may include unexplained weight loss, frequent need to urinate, blood in urine or semen, pain in lower back or hips or pelvic region(3). The risk factors for developing the Prostate cancer include – age, geography, race/ethnicity, family history, gene changes like men with lynch syndromes. Along with these there are lesser clear risk factors such as diet, obesity, smoking, prostatitis, sexually transmitted infections, vasectomy, etc.(4). Male-assigned non-binary individuals and transgender women are also at risk of developing prostate cancer (3).

Prostate Specific Antigen (PSA) tests measure the amount of PSA protein in the blood, which is an early indicator of prostate cancer. There are a few other conditions that can cause an elevated PSA level, such as benign prostatic hyperplasia (enlarged prostate) and prostatitis (prostate inflammation). Men without prostate cancer may also have positive PSA screening results (i.e., “false-positive” results). The most effective way to diagnose prostate cancer in men with positive PSA tests is to perform a trans-rectal ultrasound-guided needle biopsy of prostate tumors or lesions. PSA testing can have adverse effects on the psychological well-being due to the frequency of false-positive results (5).

In addition to digital rectal examinations, MRIs, and CT scans, prostate cancer can also be detected with other tests. The risk of prostate cancer growing and spreading is categorized according to the diagnostic grading and PSA level before the biopsy, this is known as the risk of progression. It is important to take into account the probability of progression when determining treatment and management (3).

Even though the incidence rate in India is not so high compared to other cancers, but however according to the census the mortality rate is comparatively high. The most important reason for the increase in mortality rate especially in a developing country is the lack of knowledge and awareness regarding the prostate cancer and a tool to screen the prostate cancer. As a means of reducing the mortality rates, it is crucial to raise awareness among the population about prostate cancer, screening tool for prostate cancer, and the benefits of screenings, so that prostate cancer can be diagnosed at an earlier stage for better treatment and management especially for the high risk population. This would significantly reduce the mortality rate. The main objective of this review is to find out the knowledge, awareness and practice of adult men for prostate cancer.

## Literature Search

An extensive search on Scopus, Web of Science and PubMed databases were performed on studies published between January 2000 to 2021. To find additional relevant research papers, we manually cross-checked the reference lists of all retrieved articles. The database searches used the subsequent as clinical concern heading phrases and medical text words: “Knowledge” (and) “Awareness” (and) “Practice” (and) “Screening” (and) “Prostate Cancer” (and) “English” (and) “2000:2021”.

**Figure 1:**
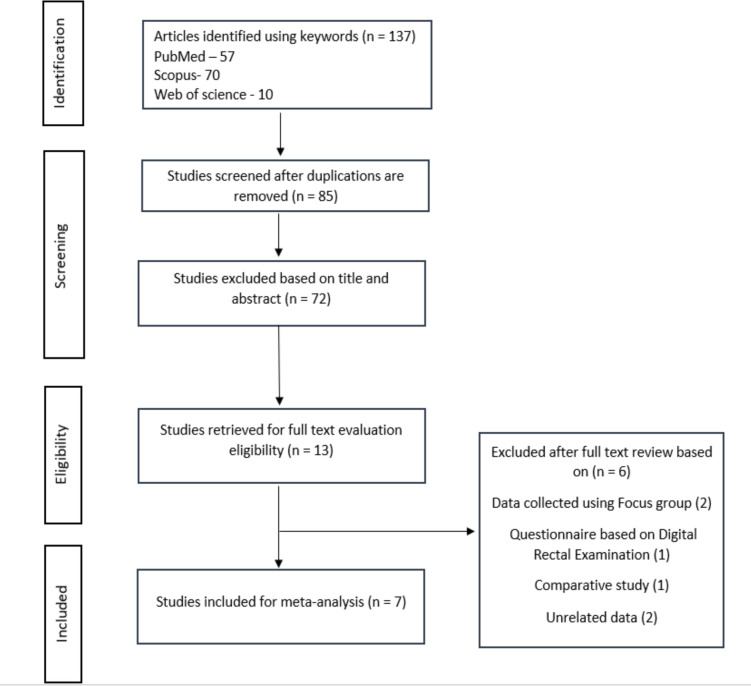
PRISMA chart depicting the search strategies and procedure for studies selection for the systematic review and meta-analysis

**Selection criteria:** The inclusion criteria considered for analyses in this study were (a) Articles dated from 2000 – 2021 (b) Published research with authentic facts in peer-reviewed journals (c) Studies published in English language only. The selection of the articles were conducted with accordance to the guidelines of the systematic reviews of the diagnostic test (Devillé et al., 2002)(6), (Campbell et al., 2019)(7). The articles underwent screening of the title and abstract fulfilling all the exclusion and inclusion standards. Based on the same inclusion and exclusion criteria's applied to the entire document, a final set of studies were included in the meta-analysis. The excluded articles were those that were published before 2000, case reports, review articles, unpublished articles were excluded as described in PRISMA Chart (Figure 1).

## Results

**Study selection and quality assessment**: The systematic seek initially yielded 137 studies, out of which 7 were covered on this meta-evaluation (Figure 1). All research studies have been published between 2000 to 2021.

**Study characteristics**: The 7 included studies involved 15854 participants within Africa and Europe. All 7 studies used cross-sectional study design. All studies have been executed in the form of a questionnaire. A detailed description of the participant's knowledge, awareness, and practice towards the screening of Prostate cancer (Table 1).

**Table 1:** Characteristics of the selected studies

Author	Year	Country	Study design	No of Participants	Age	Knowledge	Awareness	Awareness of PSA test	Screening Practice
Genevieve Benurugo	2020	Rwanda	Cross-sectional	257	>40	NA	75%	77%	45%
Asfaw N Erena	2019	Kenya	Cross-sectional	12803	15 – 54	NA	61.90%	NA	3.90%
Paraskevi A Farazi	2018	Nigeria	Cross-sectional	600	NA	NA	66.7%	NA	29.40%
Marianna Morlando	2017	Italy	Cross-sectional	625	27 – 71	82.10%	NA	72.70%	29.60%
J Sutton	2016	UK	Cross-sectional	708	NA	NA	NA	NA	NA
Belinda F. Morrison	2016	Jamaica	Cross-sectional	300	>40	84%	NA	NA	NA
Oladepo Oladimeji	2010	Nigeria	Cross-sectional	561	>50	28.70%	80%	NA	4.50%

**Figure 2:**
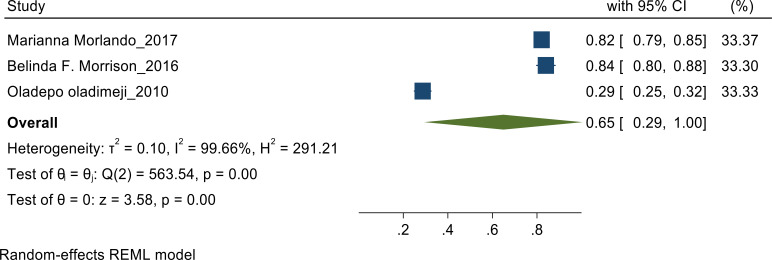
Knowledge Random-effects REML Model.

**Knowledge on prostate cancer**: Random effect meta-analysis was performed to pool the proportion of knowledge as heterogeneity found to be high (I^2^=99.6%). In this meta-analysis random effects model analysis showed that the pooled estimate of knowledge among the studies conducted by Marianna et al, Belinda F Morrison et al, and Oladepo Oladimeji et al, was found to be 65% [CI: 29%, 100%] (Figure 2)

**Awareness of signs, symptom and treatment of Prostate cancer**: Random effect meta-analysis was performed to pool the proportion of awareness as heterogeneity found to be high (I^2^=91.05%). The pooled estimate found to be 74% [CI: 66%, 82%] (Figure 3a).

**Figure 3a:**
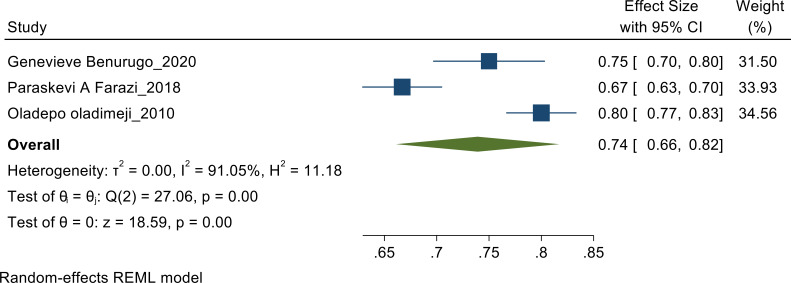
Awareness Random-effects REML Model.

**Awareness on PSA**: Fixed effect meta-analysis is performed to pool the proportion of awareness of PSA as heterogeneity found to be less (I^2^=45.56%). In this meta-analysis random effects model analysis showed that the pooled estimate of awareness of PSA among the studies conducted by Genevieve et al, Marianna et al showed the pooled estimate of 74% [CI: 71%, 77%] (Figure 3b).

**Figure 3b:**
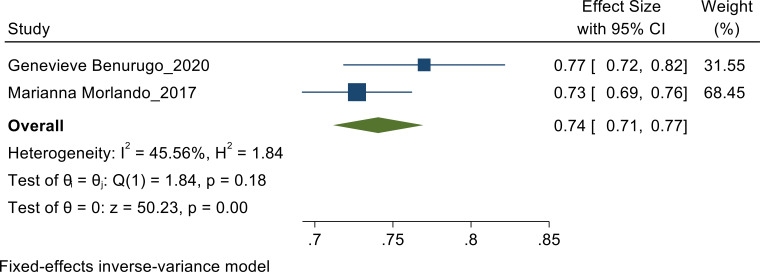
Awareness of PSA Fixed-effects inverse-variance model.

**Figure 4:**
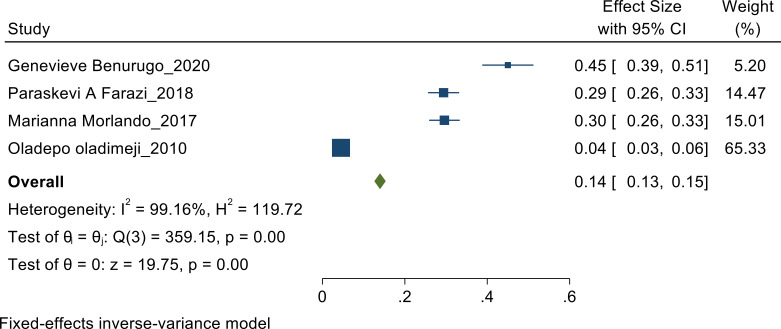
Practice Fixed-effects inverse-variance model.

**Practice of undergoing screening of prostate cancer**: Random effect meta-analysis was performed to pool the proportion of practice as heterogeneity found to be high (I^2^=99.16%). The pooled estimate found to be 14% [CI: 13%, 15%] (Figure 4).

## Discussion

In this review, the articles from the year 2000 to 2021 based on Knowledge, Awareness and Practice of screening for Prostate cancer and PSA test were included. These studies were conducted on various geographical regions like Kenya, Nigeria, Italy, UK, and Jamaica. This review identified seven articles in a span of two decades. The studies were conducted in human participants and cross-sectional study design was implemented.

According to our meta-analysis, we noted the proportion of knowledge as heterogeneity found to be high with I^2^=99.6% with a pooled estimate of 65% (Figure 2). A survey across Jamaica demonstrated a pooled estimate of 0.84(8) while the study conducted across Italy showed the pooled estimate of 0.82(9). A considerable difference was noted in the pooled estimate in the study conducted in Nigeria that showed a pool estimate of 0.29(10). The pooled estimate was higher in the studies done post 2015 than the one done in 2010 may be due to the time frame between the two studies. The study done in Jamaica demonstrated a pooled estimate of 0.84(8) which was higher while the study done in Nigeria showed the pooled estimate of 0.29(10) which was comparatively very low. The pooled estimate was higher in the studies done post 2015 than the one done in 2010 may be due to the time period when the study was conducted.

In terms of awareness and perception towards prostate cancer among men, the proportion of awareness as heterogeneity was found to be high (I^2^=91.05%). The pooled estimate found to be 74% [CI: 66%, 82%] (Figure 3a). A study was conducted across Nigeria to assess the awareness showed the pooled estimate of 0.80(10) while the study done in Rwanda showed the pooled estimate of 0.75(11) and the study done in Abuja showed a pooled estimate of 0.67(12). However, the study done in Nigeria showed higher pooled estimate than the ones done in Rwanda and Abuja.

In a study conducted by Marianna Morlando et al in Italy, the knowledge and awareness towards prostate cancer screening and PSA test was higher among the older men with higher education level, in which the PSA test has already been practiced by 29.6% of men, and 59.4% intend to do so in the future. In response to the survey, the majority of respondents had a reasonable understanding of prostate cancer and were likely to undergo the PSA test. The studies showed good awareness for PSA in the study done in Rwanda showed 0.77 pooled estimate(11) and the study done in Italy showed a pooled estimate of 0.73(9). Both the studies showed almost equal pooled estimate.

With respect to practice of screening for prostate cancer a study which was done in Rwanda showed the pooled estimate of 0.45(11), the study done in Italy showed the pooled estimate of 0.30(9), the study done in Abuja showed the pooled estimate of 0.29(12) whereas the study done in Nigeria showed the pooled estimate of 0.04(10). The study done in the Rwanda showed the highest pooled estimate for the screening practice while the study done in Nigeria showed the lowest. The studies done post 2015 had better screening practices than the one done in 2010, which may be due the time period when the studies were conducted.

Subgroup analysis was conducted for the studies done in African Continent, the study done in Rwanda showed the pooled estimate of 0.45, the study done in Abuja showed the pooled estimate of 0.29(12) whereas the study done in Nigeria showed the pooled estimate of 0.04 (10). The study done in the Rwanda showed the highest pooled estimate in African Continent for the screening practice while the study done in Nigeria showed the lowest.

However, we also noted regarding the practice of prostate cancer screening, according to the sensitivity analysis, the studies done in Rwanda showed the pooled estimate of 0.45(11) whereas the study done in Italy showed the pooled estimate of 0.30(9) and the study done in Abuja showed the pooled estimate of 0.29(12). The study done in the Rwanda showed the highest pooled estimate for the practice-sensitivity comparatively to the study done in Italy and Abuja.

Hence, it was clearly observed that the development of the tool to assess the knowledge, awareness and practice for prostate cancer is highly significant in order to increase the information on the risks, benefits of the signs and symptoms of prostate cancer and the also the screening practice of prostate cancer at an early stage.

In conclusion, it was found that there is a high level of knowledge and awareness about prostatic cancer according to the study. However, participants showed low screening practices in prostate cancer prevention, as well as a low ability to identify the risk factors and determine when prostate cancer is more likely to occur. In addition, all socio-demographic factors were strongly related to cancer screening practices. Furthermore, televisions, radios, and other mass media may not have been effective at communicating health information to men, leading to low screening rates for men. As a consequence, increasing knowledge and screening rates of prostate cancer need to be improved in the community.

Hence, the results indicate the need for developing a tool for to educate and create awareness among the men regarding the prostate cancer in both rural and urban areas in order to reduce the burden of advanced cancer by practising early screening and curb the mortality rate.
